# Development and Manufacturing of a Fibre Reinforced Thermoplastic Composite Spar Produced by Oven Vacuum Bagging

**DOI:** 10.3390/polym16152216

**Published:** 2024-08-03

**Authors:** Helena Rocha, Agnieszka Rocha, Joana Malheiro, Bruno Sousa, Andreia Vilela, Filipa Carneiro, Paulo Antunes

**Affiliations:** PIEP—Innovation in Polymer Engineering, 4800-058 Guimarães, Portugal

**Keywords:** carbon fibre-reinforced thermoplastic, thermoplastic composite, thermoplastic structural part, oven vacuum bagging, commingled fabric

## Abstract

The limited recyclability of fibre-reinforced thermoset composites has fostered the development of alternative thermoplastic-based composites and their manufacturing processes. The most common thermoplastic-based composites are often costly due to their availability in the form of prepreg materials and to the high pressure and temperatures required for their manufacturing. Yet, the manufacturing of economic and recyclable composites, made of semi-preg composite materials using traditional composite manufacturing technologies, has only been proved at a laboratory scale through the manufacturing of flat plates. This work reports the manufacturing of a real structural part, a wing spar section with complex geometry, made of commingled polyamide 12 (PA12) fibres and carbon fibres (CFs) semi-preg and by oven vacuum bagging (OVB). The composite layup was studied using finite element analysis, and processing simulation assisted in the determination of the PA12/CF preform for OVB. Processing of two forms of semi-preg materials was first evaluated and optimised. The material selection for part manufacturing was mainly defined by the materials’ processability. The spar section was manufactured in two OVB stages and was then mechanically tested. The mechanical test showed a linear strain response of the prototype up to the maximum load and validated the optimised layup configuration of the composite structure.

## 1. Introduction

Fibre reinforced polymer (FRP) composites have been extensively used in the aeronautic industry to meet the high strength and lightweight requirements of this industry [1]. For instance, a carbon fibre composite, with a density of 1600 kg/m^3^, may present a tensile strength and modulus of 1500 MPa and 200 GPa, respectively, in the fibre’s direction, while a mild steel, with a density of 7800 kg/m^3^, may present a tensile strength and modulus of 400 MPa and 208 GPa, respectively [2]. Using specific strength and specific modulus metrics, we obtain generic values of 0.05 MPa/kg·m^−3^ and 26.70 MPa/kg·m^−3^ for mild steel, respectively, and of 0.93 MPa/kg·m^−3^ and 125.00 MPa/kg·m^−3^ for carbon fibre composite, respectively. The high specific modulus obtained for carbon composites is important for applications where minimum structural weight is required combined with high stiffness.

Traditional FRP composites are made of thermoset matrices, which provide high chemical, thermal and mechanical resistance thanks to their crosslinked structure. However, the crosslinked structure of these materials hinders their recyclability. The low viscosity of uncured thermoset matrices enables the manufacturing of FRP composites at room temperature, hence at low cost [3]. For decades, inexpensive Liquid Composite Moulding (LCM) techniques, such as Resin Transfer Moulding (RTM), Hand Layup or Vacuum Assisted Resin Infusion (VARI), have been widely employed for the manufacturing of aeronautic parts, like wing spars [4,5,6,7,8,9], landing gears [9,10,11,12,13,14] or fuselage components [9,15,16,17].

The limited recyclability routes for thermoset-based composites have fostered the development of thermoplastic-based FRP composite alternatives and their manufacturing processes [18,19]. Thermoplastic-based composites present the clear advantage of being recyclable at the end of life, but the high viscosity of the polymer matrix melt requires that high temperatures and pressure are applied during processing, preventing the use of traditional composite manufacturing technologies [20]. Often, thermoplastic composite parts are made of prepreg materials, which can be very expensive. Additionally, processing of these materials requires the use of autoclaves or heated hydraulic presses for the application of high temperature and pressure to achieve the desired consolidation and mechanical properties, resulting in high energy expenditure [21]. Examples of these materials, used in aerospace applications, are polyether ether ketone (PEEK) [22], polyphenylene sulfide (PPS) [23] or polyethylenimine (PEI)-based [24] prepreg layers. The combination of these raw materials and mentioned equipment decreases processing time, as fibres are already wet by the thermoplastic matrix, and eases consolidation of the different laminas by the application of high pressure and temperature. Moreover, by enabling fast cooling rates, thermoplastics can be manufactured in short cycle times, as traditional curing and post-curing procedures are avoided, and thermoplastic parts are fully consolidated after cooling of the part [21].

Different thermoplastic composite material solutions, other than traditional prepreg material layers, have recently emerged in the form of commingled [25] fabrics or matrix powder -based fabrics [26] as more cost-effective alternatives [27]. These are semi-preg materials as the reinforcing fibres are not, or just partially, impregnated by the matrix.

Out-of-Autoclave (OoA) vacuum bagging, also referred to as oven vacuum bagging (OVB), is deemed as a lower-cost processing technology. It avoids the use of expensive autoclaves or heated press and closed moulds and uses low-cost equipment instead. The necessary equipment includes an oven, vacuum pump and a single-side open mould. The OVB technique differs from the autoclave bagging technique by the absence of positive pressure applied by the autoclave. Instead, the vacuum bagged system, reaching an absolute pressure of 10–20 mbar, is heated in an atmospheric oven up to a temperature that enables the thermoplastic matrix to become fluid enough to impregnate the reinforcing fibres. Different strategies for void reduction and improved consolidation of fibre-reinforced thermoplastic composite plates through OVB processing have been reported in the literature [28,29,30,31]. For instance, Saenz-Castillo et al. [31] demonstrated that higher temperatures are needed for improved consolidation of CF/PEEK laminates when compared to hot pressing production. Yet, the results published by Boztepe et al. [30] suggest that the temperature at which vacuum pressure is applied has a strong effect on void reduction. CF/PEEK laminates with vacuum applied at 25 °C showed decreased void content when compared to laminates with vacuum applied at 143 °C, attributed to the lower viscosity of the matrix in the latter case, leading to increased flow and closure of some air pathways arresting the gases inside the laminate and preventing them from being evacuated by the vacuum pressure. Zhang et al. [28] explored the production of CF/PEEK composite by OVB, placing a steel bar around the laminate and breather cloth in between the steel bar and the laminate to prevent pinching of the laminate at the edges upon evacuation and promote in-plane gas diffusion through the edges. It should be noted that these studies have all been conducted at the laboratory level through the manufacturing of small composite plates.

This manuscript reports the manufacturing of a thermoplastic FRP composite spar section used in an unmanned aerial vehicle (UAV) by combining the low-cost OVB processing technology with a semi-preg fabric of thermoplastic and carbon fibres (CFs). The studied part presents a complex asymmetrical geometry, which includes a bulky wedge section with enclosed foam and inserts in the composite shell, with two width changes, having different curvature radii, and a C-section. Processing parameters were first optimised for two different semi-preg fabric materials, in the form of commingled fibre fabrics (polyamide 12 (PA12)/CF) and thermoplastic powder semi-impregnated fibre fabrics (polyamide 11 (PA11)/CF), by producing flat plates by OVB. The most promising plates were cut for material characterisation, namely tensile and compression testing. PA11/CF presented superior properties in tensile and compression mode, while PA12/CF presented improved shear modulus and shear strength. Thus, the selection of one of these semi-preg fabric materials was mainly dictated by their processability. The development of the manufacturing process was supported by structural and processing finite element analysis (FEA). The composite layup configuration and overall mechanical behaviour of the part were evaluated by structural FEA, and the optimised shape of the preform to manufacture such a complex structure was conducted via numerical modelling analysis of the manufacturing process. Additionally, the FEA of the manufacturing process allowed us to identify the locations of higher distortions and stresses. After manufacturing by OVB, the spar section was mechanically tested to assess its structural integrity. The manufactured part was fixed in the two perforated inserts, while a bending load up to 310 N was applied at the extreme end of the C-section using a customisable testing apparatus. This work reports a strategy to design and produce recyclable thermoplastic composite parts used in structural applications, using a low machinery OVB manufacturing process and engineering-grade thermoplastic semi-preg composite materials. Contrarily to the existent literature, which has been testing the manufacturing of small flat plates, this work demonstrates the applicability of more cost-effective thermoplastic semi-preg composites and OVB technology for the manufacturing of a complex part used in an aeronautic application.

## 2. Materials and Methods

### 2.1. Materials

The reported part presents a complex geometry with changing cross-sections along its length, causing its height to change. Thus, semi-preg fabric materials with increased flexibility at ambient conditions were selected. These characteristics are influenced not only by the structure of the carbon fibre fabric but also by its combination with the matrix and its form. Hence, carbon fibre fabrics with a twill-weave structure were selected, as these types of structures exhibit greater drapability compared to traditional plain weave fabrics, and these were in the form of semi-impregnated materials. Since the CFs are not fully impregnated by the matrix, these materials are less rigid, allowing for the convenient stacking of several layers on the mould to produce a part with a complex geometry. This can be achieved with the thermoplastic matrix in the form of powders or fibres interwoven with the reinforcing carbon fibre (commingled fabric).

Two types of semi-impregnated materials were selected. Semi-impregnated 2/2 twill-weave CF with PA11 powder was supplied by ECC, Germany. The PA11/CF semi-preg has an areal weight of 245 g/m^2^ and fibre weight fraction of 55%, and the PA11 matrix has a melting temperature of 189 °C, as indicated by the manufacturer. Commingled semi-impregnated fibres of PA12 with 2/2 twill-weave CF were supplied by Schappe, Blyes, France. The TPFL^®^ PA12/CF semi-preg material has an areal weight of 160 g/m^2^ and fibre weight fraction of 64%, and the PA12 matrix has a melting temperature of 178 °C, as indicated by the manufacturer.

The structural polyimide foam Vespel^®^ SF-0940 (density of 500 kg/m^3^) from DuPont^TM^, Mechelen, Belgium, with longstanding high thermal resistance up to 300 °C, was used for the manufacturing of the bulky wedge section of the spar.

### 2.2. Methods

#### 2.2.1. Oven Vacuum Bagging Processing Optimisation

Aiming to minimise porosity in the composites produced by OVB, an initial study was conducted to optimise processing parameters. The processing parameters include consolidation temperature, consolidation time and vacuum pressure. The tested processing parameters were defined by taking into account a temperature/time/pressure graph provided by Schappe for processing of PA12/CF commingled semi-preg by hot press thermoforming [32]. The mentioned graph was extrapolated using a non-linear regression to find the minimum consolidation time for a specific temperature and vacuum pressure of 100 mbar, corresponding to a relative vacuum pressure of 900 mbar. The non-linear regressions for time (*t*), as a dependency of pressure (*p*), for fixed temperatures of 230 °C and 250 °C were found to be *t* = 77.835*p*^−0.994^ (coefficient of determination R^2^ = 0.999) and *t* = 62.052*p*^−1.136^ (coefficient of determination R^2^ = 0.992), respectively. The maximum temperature of 250 °C was selected to ensure suitable fluidity of the thermoplastic matrix. Table 1 presents the defined processing parameters to be tested for each material for a vacuum pressure of 100 mbar.

The thermoplastic-based composite panels were produced using the OVB process. A glass plate was used as a mould. A release film was first placed in the moulding area, where the semi-preg material layers were then placed on top, followed by a perforated release film to ease demoulding and breather fabric to spread vacuum pressure across the plates and absorb excessive matrix. The system was then sealed using sealing tape and a vacuum bag. The sealed system was placed in the oven for consolidation and connected to the vacuum hose. The vacuum pump was set to its maximum capacity of approximately 11 mbar. The temperature schedule consisted of a heating ramp at approximately 1.5 °C/min up to 70 °C, followed by an isothermal period of 30 min to check for leakages and ease consolidation, after which the temperature was increased to approximately 1.5 °C/min up to the consolidated temperature, followed by the defined isothermal period, presented in Table 1, and a final cooling stage down to ambient temperature. The OVB processing was conducted in a UFP800 oven from Memmert GmbH, Schwabach, Germany.

#### 2.2.2. Materials Characterisation

Material characterisation was performed to support material selection as well as to obtain inputs for the structural FEA simulations. All mechanical tests were performed in controlled conditions (23 ± 2 °C and 50% RH), utilising the universal testing machine Shimadzu AG-X with a 50 kN load cell. Following cross-sectional optical microscopy observations, only the specimens produced by the most promising OVB conditions were tested.

Tensile tests were conducted according to the International ASTM D3039 standard [33] at a speed of 2 mm/min. Specimens with fibre orientation of 0°/90° and −45°/+45° were prepared with approximate dimensions of 250.0 × 25.0 × 2.5 mm. Five specimens were tested for each condition. The specimens with fibres oriented at 0°/90° were used to determine the elastic modulus (*E*), Poisson ratio (ν) and ultimate stress (X_t_), according to ASTM D3039 standard, while the specimens with fibres oriented at −45°/+45° were used for determination of shear modulus (G_12_) and ultimate shear stress (τ_12_), according to the International ASTM D3518 standard. The samples were instrumented with strain gauges (SGT-3/350-XY41 from OMEGA Engineering Inc., Manchester, UK). The installation of the strain gauges followed the procedure recommended by the supplier. Their correct installation was verified by measuring their resistance, with only a ±0.3% deviation allowed, and by ensuring the continuity of the electrical circuit.

Compression tests were conducted according to the SACMA SRM 1R94 standard [34] at a speed of 1 mm/min. Five specimens with approximate dimensions of 80.0 × 15.0 × 3.0 mm were produced with fibres oriented at 0°/90° to determine the compressive strength (X_c_).

Cross-sectional optical microscopy observations were conducted for microstructural analysis in the InfiniteFocusSL optical microscope from Alicona, Graz, Austria.

#### 2.2.3. Simulation of Manufacturing Process of Thermoplastic Composite Part

PAM-Form^TM^ software, version 18.0.1, from ESI Group was used as a manufacturing process simulation tool to determine the optimised shape of the preform and identify the regions with higher distortions and stresses.

For the simulation of the OVB manufacturing process, the conformation was considered to be similar to that of a dry reinforcement, i.e., the dependence on the thermal conditions was neglected. Taking into account the complex geometry of the part, the numerical simulation modelled the conformation of the outer shell laminate only, on which all deformation of the laminate occurs. The optimisation of the preform geometry was performed by starting with a simple geometry (rectangular shape). Then, by successive iterations, the preform geometry was modified depending on the previous deformation results until an optimised preform geometry was obtained, i.e., a preform geometry that results in less localised distortion, fewer wrinkles, lower stresses and no material excess.

**Figure 1:**
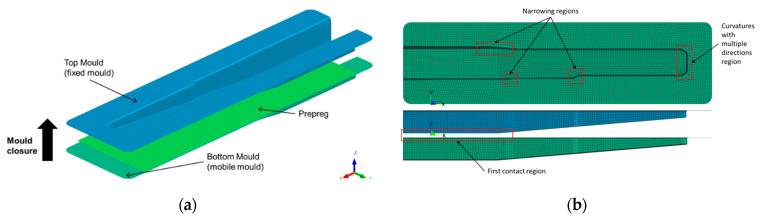
Processing simulation model: (**a**) vertical press machine approach; (**b**) composite part geometry.

The model was built according to a vertical press machine approach: a rigid mould and counter-mould were considered; the top mould was fixed in all directions; the prepreg was placed on the bottom mould; several fixing points were defined to maintain the prepreg positioned in place; and the bottom mould moved up to close (normal to mould surface direction), with a constant speed (14.26 mm/s). The conformation of the prepreg is performed in one step, in which the mould displacement occurs in a single movement. The effect of gravity was neglected. The moulding tools and material were designed as surfaces in the processing simulation model. This approach allows the building of simpler meshes with 2D elements. A schematic representation of the processing simulation model is presented in Figure 1.

The moulds are defined as a generic rigid metal (steel) with the following properties: density (ρ) of 7.8 g/cm^3^; Young’s modulus (*E*) of 210 GPa; and Poisson’s ratio (ν) of 0.3. The characteristics of the PA12/CF prepreg are summarised in Table 2, while the mechanical properties are in Table 3. The contact between prepreg and mould material was defined by a constant coefficient of friction of 0.17, characteristic of dry fabric prepregs in contact with a metal [35,36].

#### 2.2.4. Structural Simulation of Thermoplastic Composite Part

Numerical simulations were performed in order to validate the layup configuration of the composite material in the spar section, using Abaqus software, version 2020, from Dassault Systèmes. The geometry of the composite was discretised by linear shell elements (S4 and S3), while foam, loading block and inserts were modelled by solid elements (C3D4 and C3D5), with an average size of 4 mm. The aluminium inserts and loading block, as well as the foam, were modelled as isotropic materials and their density, Young’s modulus, Poisson’s ratio and σ_y_ (yield stress and ultimate strength of the aluminium and foam, respectively) are presented in Table 4. The composite was modelled as a linear material with a lamina type. The mechanical properties of the PA12/CF composite used for the composite part were obtained from the mechanical characterisation tests and are presented in Table 5. The density was taken from the material technical datasheet (1480 kg/m^3^).

**Figure 2:**
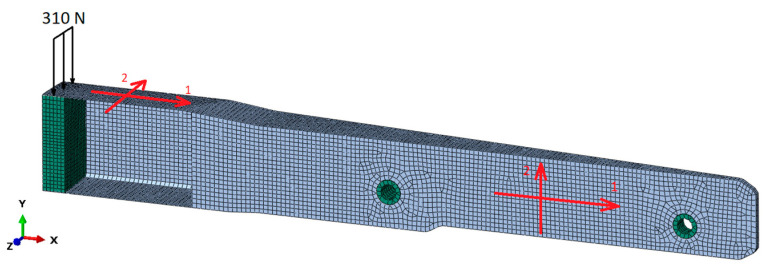
Numerical model of the spar. Composite in grey with the orientation of the fibres (1 and 2 corresponding to 0° and 90°) and aluminium loading block and inserts in green. Load of 310 N applied to the spar extremity.

The connection between the composite and the foam and loading block, as well as between the foam and the metallic inserts, was modelled as a tie constraint. The degrees of freedom (DOF) in the displacement of the inserts’ internal surfaces were considered to be completely constrained. A static load of 310 N was applied to the free extremity of the spar. The FEA simulation model is schematically presented in Figure 2.

#### 2.2.5. Manufacturing of Thermoplastic Composite Part

**Figure 3:**
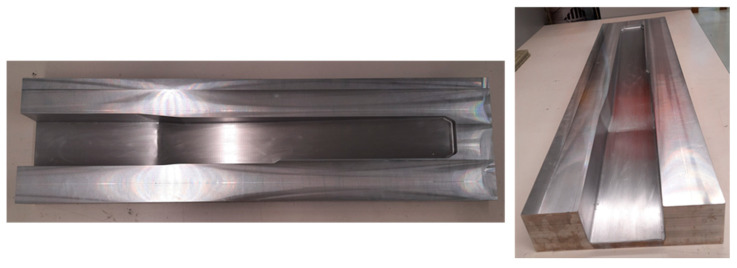
Open aluminium mould for manufacturing of a spar partition with a bulky wedge and C-section by OVB.

The manufacturing of the thermoplastic composite spar part section was conducted by the OVB process, using an open mould made of aluminium, shown in Figure 3. The mould has a brim all around the part cavity, approximately 60 mm, for the placement of sealing tape and a vacuum bag.

**Figure 4:**
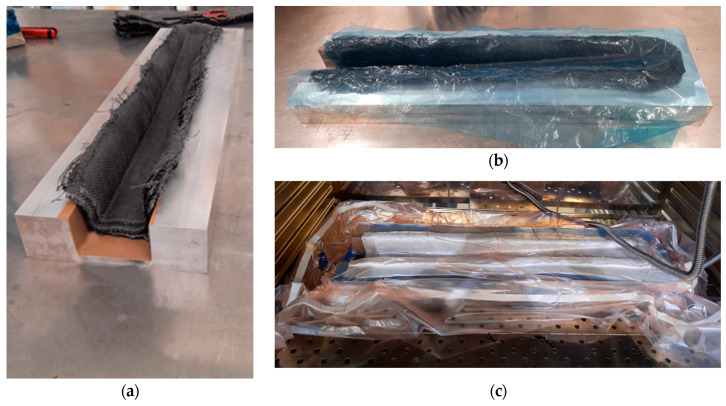
First stage of consolidation by OVB: (**a**) stacking of PA12/CF semi-preg layers over the mould; (**b**) placement of perforated film; and (**c**) sealed vacuum bag system for consolidation in the oven.

According to the conducted structural FEA, as described in Section 2.2.4, the results of which are presented in Section 3.3, the spar section should have a thickness of 3.3 mm, composed of 25 layers of the PA12/CF composite. Due to the impossibility of stacking such a large number of layers, which have a significant thickness prior to their consolidation, together with the foam in the mould cavity, the part was produced in two stages. Initially, the 25 layers of semi-preg material making the outer shell of the part (bottom and sides) were stacked in the mould. The moulding surface was covered by polytetrafluoroethylene (PTFE) adhesive film to ease demoulding. Cutting of these layers followed the preform optimisation resultant from the simulation of the manufacturing process, described in Section 2.2.3, which results are discussed in Section 3.2. Figure 4 presents photographs of this first stage of the manufacturing process.

**Figure 5:**
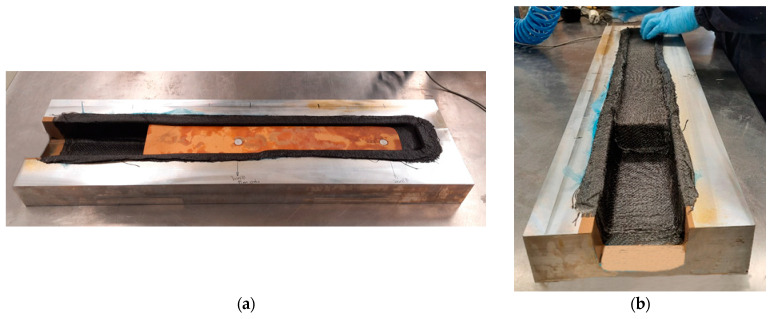
(**a**) Structural foam and inserts placed in the cavity to form the bulky section of the spar; (**b**) coverage of the structural foam to produce the spar section with a bulky wedge.

After consolidation and cooling of the first section, the structural foam was machined and polished to fit the cavity of the part, the metallic inserts were fitted into it, and all were introduced in the moulded cavity (see Figure 5a). The foam was then covered by 25 layers of semi-preg material, as shown in Figure 5b. The second vacuum bagging process followed the procedure described above. Both consolidation stages consisted of an isothermal period of 30 min at 70 °C, followed by a second one of 135 min at 230 °C. Following the second consolidation stage and cooling, the part was demoulded, trimmed and polished.

#### 2.2.6. Mechanical Testing of Thermoplastic Composite Part

**Figure 6:**
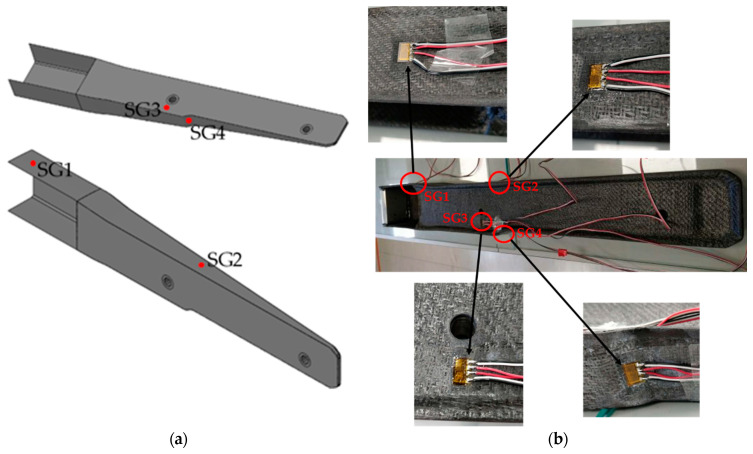
(**a**) Schematic representation and (**b**) real locations of strain gauges in the spar section part.

The produced prototype was mechanically tested to evaluate its integrity when subjected to a load. Furthermore, this mechanical test aimed to validate the composite layup configuration deduced from the structural FEA. For that, the thermoplastic composite part was instrumented with bidirectional strain gauges (SGT-3/350-XY41 from OMEGA Engineering Inc., Manchester, UK) to monitor strain at four critical points: (SG1) close to the load application area; (SG2) at the middle of the lateral side of the bulky section; (SG3) close to the screwing zone, which was identified in the numerical simulations as a critical area (see Section 3.3); and (SG4) at the lateral slope area of the bulky section, opposite to SG2, as observed in Figure 6. The cDAQ-9174 data acquisition system, from National Instruments, Austin, TX, USA, was used to record the strain gauge data at a sampling rate of 1000 Hz.

**Figure 7:**
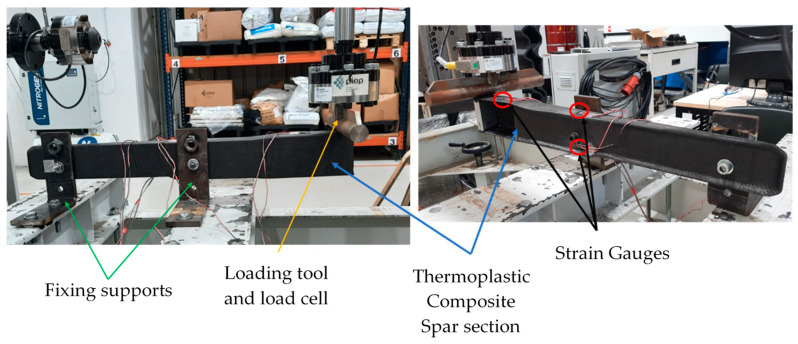
Finalised section of the spar part assembled in the mechanical testing apparatus.

The part was fixed in the two perforated inserts, while the bending load was applied in the bonded 2 cm thick aluminium block at the end of the C-section (see Figure 7). The aluminium block enables the distribution of the applied load along a 2 cm wide extension of the C-section. The bending tests were initiated with a pre-load of 5 N, and the load was then increased up to 310 N at a speed of 0.01 kN/s using a customisable testing apparatus with a 50 kN load cell (Figure 7).

## 3. Results

### 3.1. Materials Characterisation

**Figure 8:**
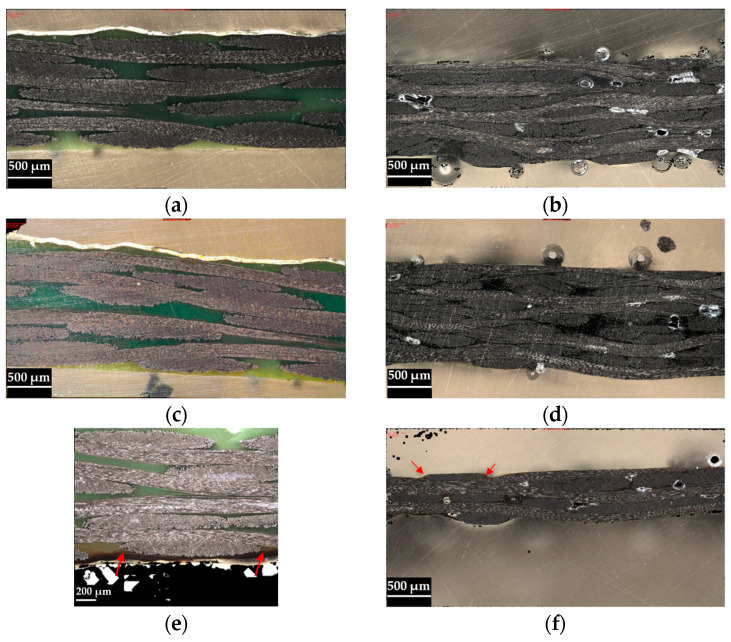
Optical micrographs of the composite plates cross-section: (**a**) PA11/CF produced at 230 °C during 86 min; (**b**) PA12/CF produced at 230 °C during 86 min; (**c**) PA11/CF produced at 230 °C during 135 min; (**d**) PA12/CF produced at 230 °C during 135 min; (**e**) PA11/CF produced at 250 °C during 70 min; (**f**) PA12/CF produced at 250 °C during 70 min.

Figure 8 presents images of both types of laminates obtained from optical microscopy observations. It can be noticed that plates made of PA11/CF do not present porosity, which is not the case for plates made of PA12/CF. This can be attributed to the higher volumetric fraction of the matrix in the PA11/CF composite, which eases consolidation. The higher consolidation temperature of 250 °C did not significantly influence the porosity content but led to smaller pores. The pore size in the specimens manufactured at 230 °C had a few voids with length up to 860 µm, while specimens manufactured at 250 °C had voids up to 366 µm in length. However, the higher processing temperature caused thermal degradation of the resin, identified by discoloured regions (indicated by red arrows in Figure 8e,f), which might have been caused by the long exposure of the materials to this temperature. This is readily observable in the PA11/CF specimens (Figure 8e) due to the higher resin fraction, while this is only observed in small resin-rich areas of the PA12/CF (Figure 8f), with the high CF content providing increased thermal resistance. A significant improvement could not be found in the specimens consolidated at 230 °C for 135 min when compared to the specimens consolidated for 86 min. Nevertheless, even this longer consolidation period can be faster than the curing process of thermoset composite counterparts.

As can be seen in Table 5, PA11/CF presents superior properties in tensile and compression, while PA12/CF presents improved shear modulus and strength. The reason for these results cannot be directly attributed to the presence of voids in the PA12/CF since the full disclosure of the carbon fibres properties in the distinct semi-preg materials is not disclosed by the different suppliers. Yet, as expected, the increased resin content in the PA11/CF impaired its shear properties when compared to PA12/CF. Moreover, during the fabrication of the PA11/CF plates, it was possible to observe that the PA11/CF fabric is not flexible and has inferior drapability due to partial impregnation of the fibres by the resin in the form of powder. An additional increase in the number of layers of this material might make the production of the final product difficult or even impossible.

### 3.2. Simulation of Manufacturing Process of Thermoplastic Composite Part

**Figure 9:**
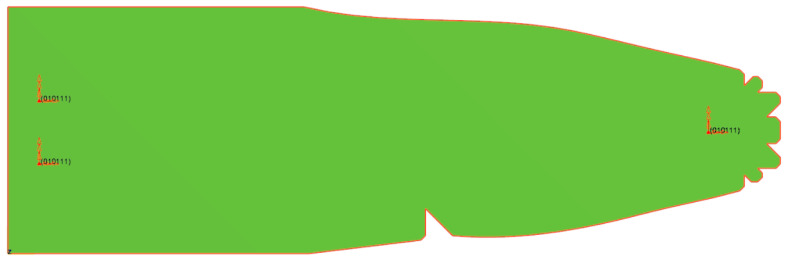
Optimised preform geometry with fixation points.

The optimised geometry of the preform is depicted in Figure 9.

**Figure 10:**
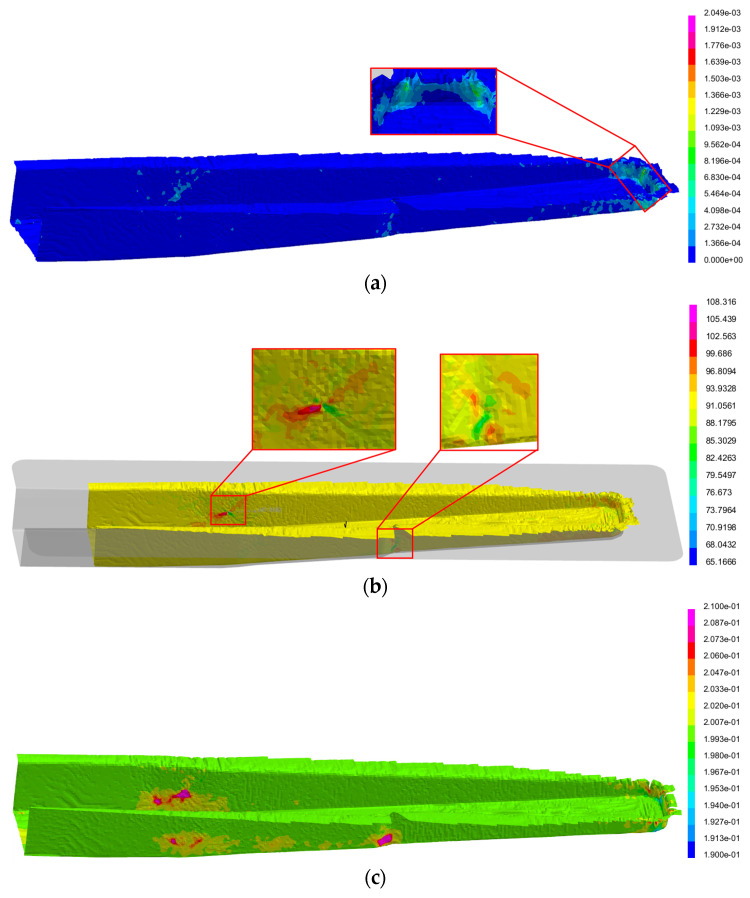
Conformation results obtained by the simulation of the optimised preform: (**a**) total stress distribution (GPa); (**b**) fibre angle distribution (°); (**c**) thickness variation (mm/ply).

The simulation results for the optimised preform geometry are summarised in Table 6 and illustrated in Figure 10. The results show that the maximum total stress and maximum shear stress are limited to the narrow edge of the part, where the prepreg is bent in multiple curvatures (Table 6 and Figure 10a), with a magnitude equal to 2.049 MPa and 0.987 MPa, respectively. Yet, these values are considerably lower than the maximum tensile and shear stresses of the material (Table 3). The distortion of the original 90° angle occurs in the narrowing regions near the widest and deepest part of the geometry, which concurs with the region of the first contact between mould and prepreg, within a range of 65.17° and 108.31° (Table 6 and Figure 10b). The thickness distribution (Figure 10c) shows that it is the narrowing regions that suffer the greatest thickness variation per layer (±0.01 mm, Table 6) as a consequence of the high distortion observed in these regions. Thus, despite significant variations from the original angle with deformation (±20° from the initial angle, Table 6), the numerical predicted stresses are considerably lower than the maximum stresses of the material (Table 3 and Table 6). These results suggest that forming does not result in damage to the fibrous reinforcement nor in significant defects.

### 3.3. Structural Simulation of Thermoplastic Composite Part

**Figure 11:**
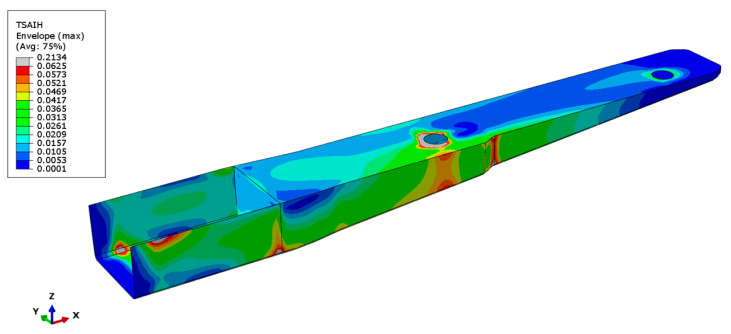
Distribution of Tsai–Hill criterion in the composite subjected to the 310 N loading. Please note that the scale was limited to 0.0625, which corresponds to a safety factor of 4. The grey-coloured areas present regions where the safety factor is below 4.

The optimised laminate, which can sustain a load of 310 N with a safety factor of 4, consists of 25 layers of the PA12/CF semi-preg oriented at 0°/90°, resulting in a total thickness of 3.3 mm. Results of the structural simulation of the spar subjected to 310 N loading are presented below. The distribution of the Tsai–Hill failure criterion can be seen in Figure 11. The safety factor is not achieved in areas close to the metallic insert that have displacement DOF constrained and close to the area where the loading is applied. The increase in stress in those areas is expected due to applied constraints but is not significant.

**Figure 12:**
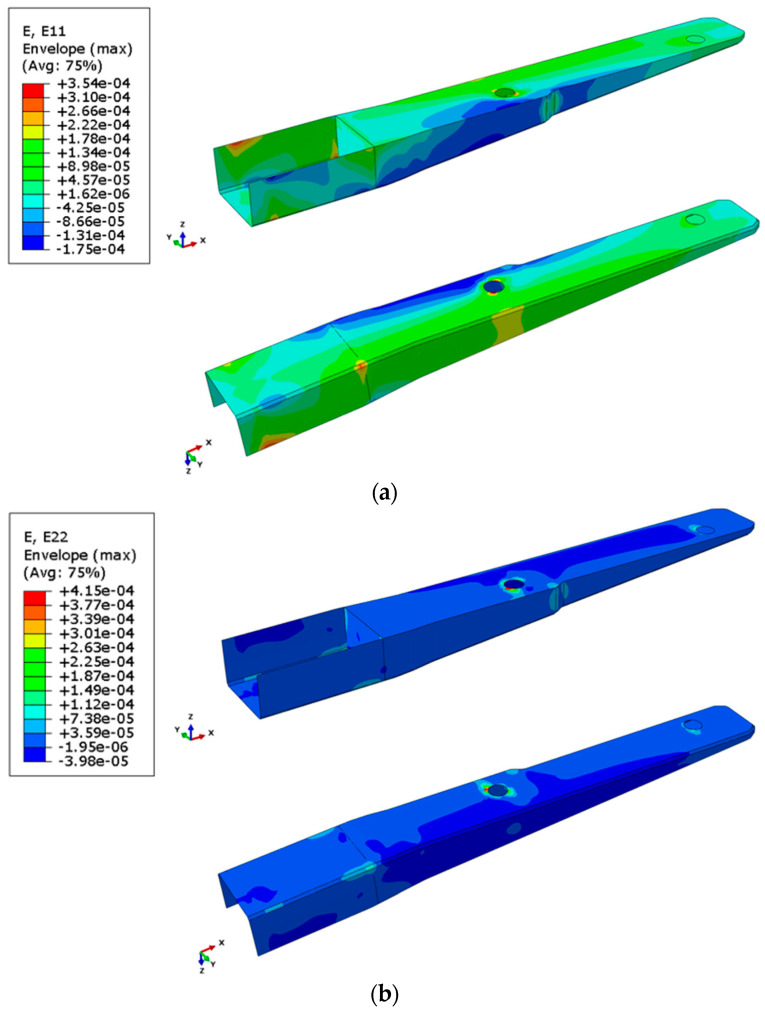
Distribution of strains in the spar: (**a**) longitudinal strain; (**b**) transversal strain.

Figure 12 presents the distribution of longitudinal (E_11_) and transversal (E_22_) strains in the spar. As can be expected, the laminate in the bottom part of the spar is subjected to compression loading in the longitudinal direction, while the top part is subjected to tensile forces.

### 3.4. Manufacturing of the Thermoplastic Composite Part

**Figure 13:**
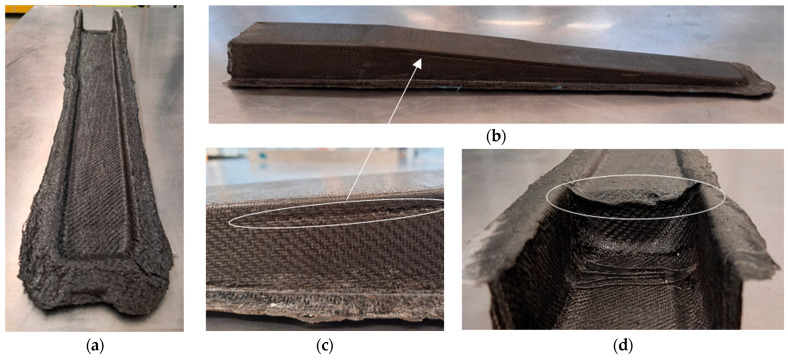
Demoulded part in (**a**,**b**), where it is possible to see some wrinkles resultant from OVB process in (**c**,**d**).

Wrinkles appeared near the bending zone of the C-section (see Figure 13c) and at the transition between the foam-containing zone and the C-section (see Figure 13d) (identified by white ellipses). These wrinkles result from the high number of layers in the part and the compressive force applied by the vacuum bag, distributed along the part, which slightly pulls the layers of semi-preg towards the interior of the cavity in the C-section. In the case of the wrinkles shown in Figure 13c, these may also be attributed to an incorrect manual stacking process of the bottom layers. Nevertheless, the part presented high stiffness, which was validated in the mechanical tests presented in Section 3.5.

**Figure 14:**
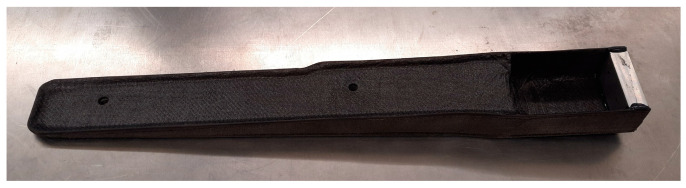
Finalised section of the spar part with drilled inserts and aluminium accessory for mechanical testing.

Figure 14 presents the finished manufactured thermoplastic composite spar section, with perforated inserts and aluminium accessories, for mechanical testing.

### 3.5. Mechanical Testing of Thermoplastic Composite Part

**Figure 15:**
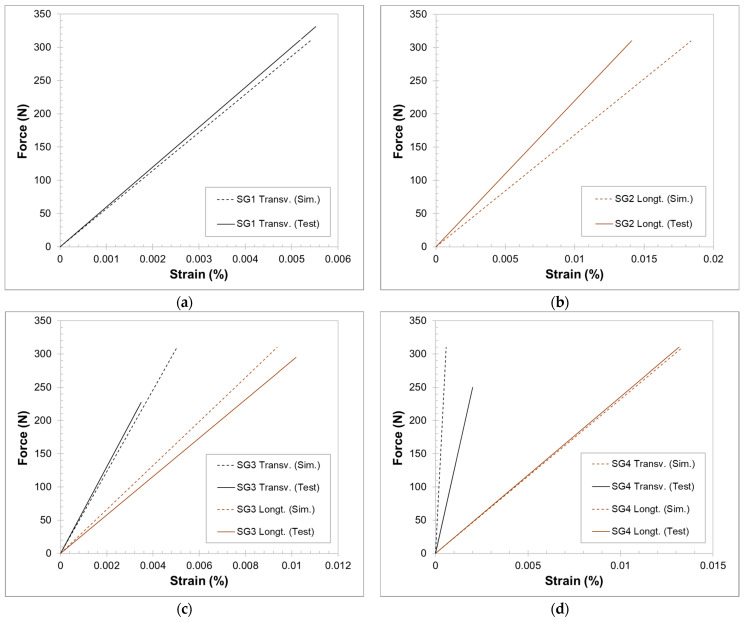
Comparison of strain measured during mechanical testing of the prototype with simulated values: at locations presented for strain gauges 1–4 ((**a**–**d**), respectively), in the longitudinal (along the length of the spar section) and transverse direction. Note that the presented values for SG3 in the longitudinal direction and SG4 in the transverse direction are compressive strains.

The strain measured during mechanical testing by the different strain gauges is presented in Figure 15. However, it should be noted that there was an issue associated with strain gauges 1 and 2 (SG1 and SG2), which could only provide data readings in only one direction. This may be attributed to saturation or damage during the installation of the part in the test rig. Nevertheless, the strain gauges exhibited reliable data in the other direction. The graphs present the strain in both longitudinal and transverse directions, the first in the direction of the length and the latter in its transverse direction, i.e., along the height of the spar section. Although the load was linearly increased up to 310 N, some strain gauges failed before that. Additionally, the strain obtained in the structural simulation for the elements in the area of each strain gauge is also presented for comparison.

The strain values obtained from the structural FEA present the same order of magnitude as the experimental strain measured by the strain gauges. These data confirm and validate the conducted structural simulations for the optimisation of the composite layup. Moreover, looking at the experimental measurements, it can also be concluded that the part was not loaded beyond its elastic region.

The minor discrepancies observed between numerical and experimental data may arise from challenges in accurately mapping the true positions (and coverage areas) of the strain sensors within the discretised domain. This challenge is a result of the irregular geometrical configuration in the mesh, particularly in certain zones of the model.

## 4. Discussion

The utilisation of fibre-reinforced thermoplastic composites at a real industrial scale in the aeronautic industry, namely, for the manufacturing of UAV structural parts, can be economically satisfied with the combination of thermoplastic/CF semi-preg materials and lower-cost manufacturing technologies, such as OVB, where lower pressures are involved. This could bring evident advantages to the recycling routes at the end of the life of such structures. Mechanical recycling is a possibility, where the disposed thermoplastic composite part can be shredded and new parts can be moulded by hot pressing. Alternatively, the part can be micronised and compounded with compatible thermoplastic polymers and additives to be used on other polymer manufacturing technologies, such as injection moulding or extrusion.

This paper demonstrates the feasibility of manufacturing a highly complex structural part, resorting to commingled PA12 fibres and CF semi-preg and OVB processing. The manufacturing parameters, including consolidation temperature and time, were first studied at the plate level, considering the maximum vacuum level capability of the available vacuum pump, approximately 11 mbar. Given the improved processability of commingled PA12/CF semi-preg, despite its slightly inferior tensile properties when compared to PA11/CF semi-preg, the commingled PA12/CF semi-preg was selected to produce the spar section. Since the carbon fibres in the commingled PA12/CF semi-preg are not impregnated by the PA12 matrix, and the carbon fibres in the PA11/CF semi-preg are partially impregnated by the PA11 matrix powder, the commingled PA12/CF semi-preg presents high flexibility, allowing to produce such a complex structural part. However, the manufacturing of these materials through OVB yields inferior mechanical properties when compared to traditional epoxy/twill carbon fibre composites. The literature has reported elastic moduli in the range of 55–57.8 GPa [37,38] for epoxy composites, which represents a difference of about 21% and 70%, when compared to the reported PA11/CF (E_1_ = E_2_ = 45.27 ± 2.72 GPa) and PA12/CF (E_1_ = E_2_ = 32.30 ± 8.47 GPa), respectively.

Finite element analyses of the manufacturing process were conducted to determine the optimised preform to produce the spar section with minimal wrinkles. Additionally, structural FEA was performed to find the minimum thickness of the semi-preg composite material to withstand the required load level. According to the results of the structural FEA, the part should have a thickness of 3.3 mm, which was achieved by stacking 25 composite layers. In terms of the areal weight of carbon fibre, the part section reported in this study is fairly comparable to its traditional counterpart made of thermoset-reinforced CF composite, with a total areal weight of about 2500 g/m^2^.

Given the manual labour and the large number of semi-preg layers in the thermoplastic composite laminate, the manufactured part presents some wrinkles associated with the manual pressing of the semi-preg layers and the suction effect of the vacuum bag. Regardless of such defects and of the likely presence of porosities, as observed by optical microscopy in the plate laminates, the thermoplastic composite spar section prototype revealed a linear strain response up to 0.014% at the maximum load of 310 N. The measured strain values were found to be in the same order of magnitude as that of the structural FEA, which validates the optimised composite layup configuration.

Although the thermoplastic composite application demonstration reported here was conducted on a real composite structure, its geometry could be redesigned to allow different manufacturing strategies to avoid two OVB processing stages, fostering industrialisation. Moreover, the OVB process allows for a cleaner process, preventing any contact of the technicians with the liquid resin while preparing the wet layup manufacturing system. The industrialisation of the materials and process reported here would require minimal investments to convert a wet layup manufacturing facility to an OVB manufacturing setup, where vacuum pumps are already available and, possibly, also ovens that can be heated up to 250 °C.

Future developments would encompass the manufacturing of the full spar part, mechanical testing, including static and fatigue testing, and exploitation of recycling routes.

## Figures and Tables

**Table 1:** Tested processing parameters of CF/PA semi-preg materials by OVB at a vacuum pressure of 100 mbar.

Experience	Consolidation Temperature (°C)	Consolidation Time (min)
1	230	86
2	230	135
3	250	70

**Table 2:** Characteristics of the commingled prepreg TPFL^®^ PA12/CF, with 2 × 2 twill-weave pattern.

Property	Value
Total Areal Weight (g/m^2^)	160
Fibre Areal Weight (g/m^2^)	102
Fibre Volume Fraction (%)	50
Thickness (mm)	0.20
Yarn Density (warp × weft/cm)	8 × 8
Density of Composite (g/cm^3^)	1.48

**Table 3:** Mechanical properties of the commingled prepreg TPFL^®^ PA12/CF, with 2 × 2 twill-weave pattern.

Fibre Direction	Initial Angle Between Fibres (°)	Maximum Strength of the Material (MPa)	Bending Stiffness (Pa)	Maximum Shear Strength of the Material (MPa)
0°	90	22.4	620	8.78
90°		21.5	585	

**Table 4:** Mechanical properties of the aluminium and SF-0940 foam used in the structural simulation.

Property	Aluminium	SF-0940 Foam
ρ (kg/m^3^)	2750	500
*E* (MPa)	70,000	521.2
ν	0.33	0.4
σ_y_ (MPa)	250	25

**Table 5:** Mechanical properties of the laminates PA11/CF and PA12/CF, manufactured by OVB at 230 °C for 135 min.

	E_1_ = E_2_ (GPa)	ν_12_	X_t_ (MPa)	Xc (MPa)	G_12_ (GPa)	τ_12_ (MPa)
PA11/CF	45.27 ± 2.72	0.08 ± 0.03	362.61 ± 17.20	194.6 ± 18.8	1.43 ± 0.08	45.64 ± 3.31
PA12/CF	32.30 ± 8.47	0.08 ± 0.04	357.94 ± 16.15	114.9 ± 4.3	2.06 ± 0.13	51.64 ± 1.21

**Table 6:** Results for the conformation of the preform optimised geometry of the prepreg TPFL^®^ PA12/CF with 2 × 2 twill-weave pattern.

Total Maximum Stress (MPa)	Location	Total Maximum Shear Stress (MPa)	Location	Angle Variation (°)	Thickness Variation (mm/ply)
2.049	Narrow edge	0.987	Narrow edge	[65.17; 108.31]	[0.19; 0.21]

## Data Availability

The original contributions presented in the study are included in the article, further inquiries can be directed to the corresponding author/s.
